# Defective jagged-1 signaling affects GnRH development and contributes to congenital hypogonadotropic hypogonadism

**DOI:** 10.1172/jci.insight.161998

**Published:** 2023-03-08

**Authors:** Ludovica Cotellessa, Federica Marelli, Paolo Duminuco, Michela Adamo, Georgios E. Papadakis, Lucia Bartoloni, Naoko Sato, Mariarosaria Lang-Muritano, Amineh Troendle, Waljit S. Dhillo, Annamaria Morelli, Giulia Guarnieri, Nelly Pitteloud, Luca Persani, Marco Bonomi, Paolo Giacobini, Valeria Vezzoli

**Affiliations:** 1 Department of Medical Biotechnology and Translational Medicine, University of Milan, Milan, Italy.; 2 University Lille, INSERM, CHU Lille, Laboratory of Development and Plasticity of the Neuroendocrine Brain, Lille Neuroscience & Cognition UMR-S 1172, FHU 1000 days for health, Lille, France.; 3 Department of Endocrine and Metabolic Diseases, IRCCS Istituto Auxologico Italiano, Milan, Italy.; 4 Department of Endocrinology, Diabetology, and Metabolism, Lausanne University Hospital (CHUV), Lausanne, Switzerland.; 5 Department of Pediatrics, University of Tokyo Graduate School of Medicine, Tokyo, Japan.; 6 Department of Pediatric Endocrinology and Diabetology, University Children’s Hospital, Zurich, Switzerland.; 7 Department of Endocrinology, Diabetology, and Metabolism, Lindenhofspital, Bern, Switzerland.; 8 Division of Diabetes, Endocrinology and Metabolism, Imperial College London, London, United Kingdom.; 9 Department of Experimental and Clinical Medicine, University of Florence, Italy.

**Keywords:** Development, Genetics, Fertility, Genetic diseases, Neurodevelopment

## Abstract

In vertebrate species, fertility is controlled by gonadotropin-releasing hormone (GnRH) neurons. GnRH cells arise outside the central nervous system, in the developing olfactory pit, and migrate along olfactory/vomeronasal/terminal nerve axons into the forebrain during embryonic development. Congenital hypogonadotropic hypogonadism (CHH) and Kallmann syndrome are rare genetic disorders characterized by infertility, and they are associated with defects in GnRH neuron migration and/or altered GnRH secretion and signaling. Here, we documented the expression of the jagged-1/Notch signaling pathway in GnRH neurons and along the GnRH neuron migratory route both in zebrafish embryos and in human fetuses. Genetic knockdown of the zebrafish ortholog of JAG1 (*jag1b*) resulted in altered GnRH migration and olfactory axonal projections to the olfactory bulbs. Next-generation sequencing was performed in 467 CHH unrelated probands, leading to the identification of heterozygous rare variants in *JAG1*. Functional in vitro validation of *JAG1* mutants revealed that 7 out of the 9 studied variants exhibited reduced protein levels and altered subcellular localization. Together our data provide compelling evidence that Jag1/Notch signaling plays a prominent role in the development of GnRH neurons, and we propose that JAG1 insufficiency may contribute to the pathogenesis of CHH in humans.

## Graphical abstract

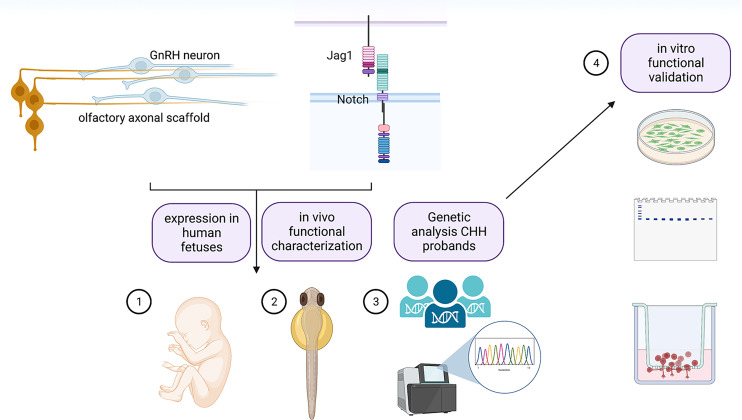

## Introduction

Reproduction and fertility in mammals are strictly dependent on a small population of hypothalamic neurons, the gonadotropin-releasing hormone (GnRH) neurons. GnRH-secreting neurons are unique neuroendocrine cells as they originate in the nasal placode, outside the central nervous system, during embryonic development, and migrate to the hypothalamus along the vomeronasal nerves (VNNs) and terminal nerves (TNs) (1). This process is evolutionarily conserved and follows a similar spatiotemporal pattern in all mammals (2), including humans (3, 4).

Any misfunction in the GnRH system leads to a human genetic disorder called congenital hypogonadotropic hypogonadism (CHH) (5). When CHH associates with hypo/anosmia, the disease is known as Kallmann syndrome (KS) (6, 7). In approximately 50% of affected individuals, no mutations can be identified in the known CHH genes, indicating that the genetics underlying CHH is still largely unknown (6, 8).

The GnRH migratory process is orchestrated by a plethora of factors, expressed along the GnRH migratory route, controlling cell signaling, adhesion, motility, and neurite and axonal elongation (8, 9).

It has been previously reported that Notch1 is expressed in the developing mouse olfactory epithelium from embryonic day 11 (10–12), corresponding to the beginning of the GnRH migratory process and olfactory axonal targeting (2). More recently, a group demonstrated an essential role for Notch1 in the development of the vomeronasal organ (13), where GnRH neurons are born (2, 4). However, the specific role of Notch signaling in the development of the GnRH system has not been investigated to our knowledge.

Four vertebrate Notch genes have been identified and are designated Notch1, Notch2, Notch3, and Notch4 (14–24). Mammalian Notch genes are widely expressed during embryonic development, suggesting that Notch regulates the differentiation of many different cell types.

Signaling is initiated when a Notch receptor on one cell interacts with Notch ligands, such as Delta-like ligand–1 (Dll-1), -3, and -4, and Serrate-like ligands (Jagged-1 and -2), on an adjacent cell (25).

Pathogenic allelic variants in *JAG1* gene have been also described as causing Alagille syndrome, a rare autosomal dominant disease with characteristic liver, cardiac, eye, vertebral, and facial phenotypes (26).

In this study, we document for the first time to our knowledge the expression of *JAG1*, *DLL1*, and *NOTCH1–4* genes in the human GnRH and olfactory/vomeronasal system during early fetal development.

We show that genetic invalidation of the zebrafish ortholog of *JAG1* (*jag1b*) results in altered GnRH migration and olfactory axonal projections to the olfactory bulb. Moreover, we provide evidence that pharmacological invalidation of the Notch signaling pathway impairs the motility of a murine immortalized GnRH cell line and of human fetal GnRH-secreting neuroblasts representative of developing GnRH neurons (FNCB4) (27, 28).

The involvement of the JAG1 signaling pathway in GnRH development led to the identification of 9 heterozygous mutations in *JAG1* among 467 patients with CHH. Collectively, this study identified a potentially novel embryonic role of Jag1/Notch signaling in the development of GnRH neurons and provided genetic evidence that disturbance of this signaling can contribute to CHH phenotype in humans.

## Results

### JAG1 and NOTCH paralog mRNAs are expressed in and along the GnRH migratory route in human fetuses.

**Figure 1:**
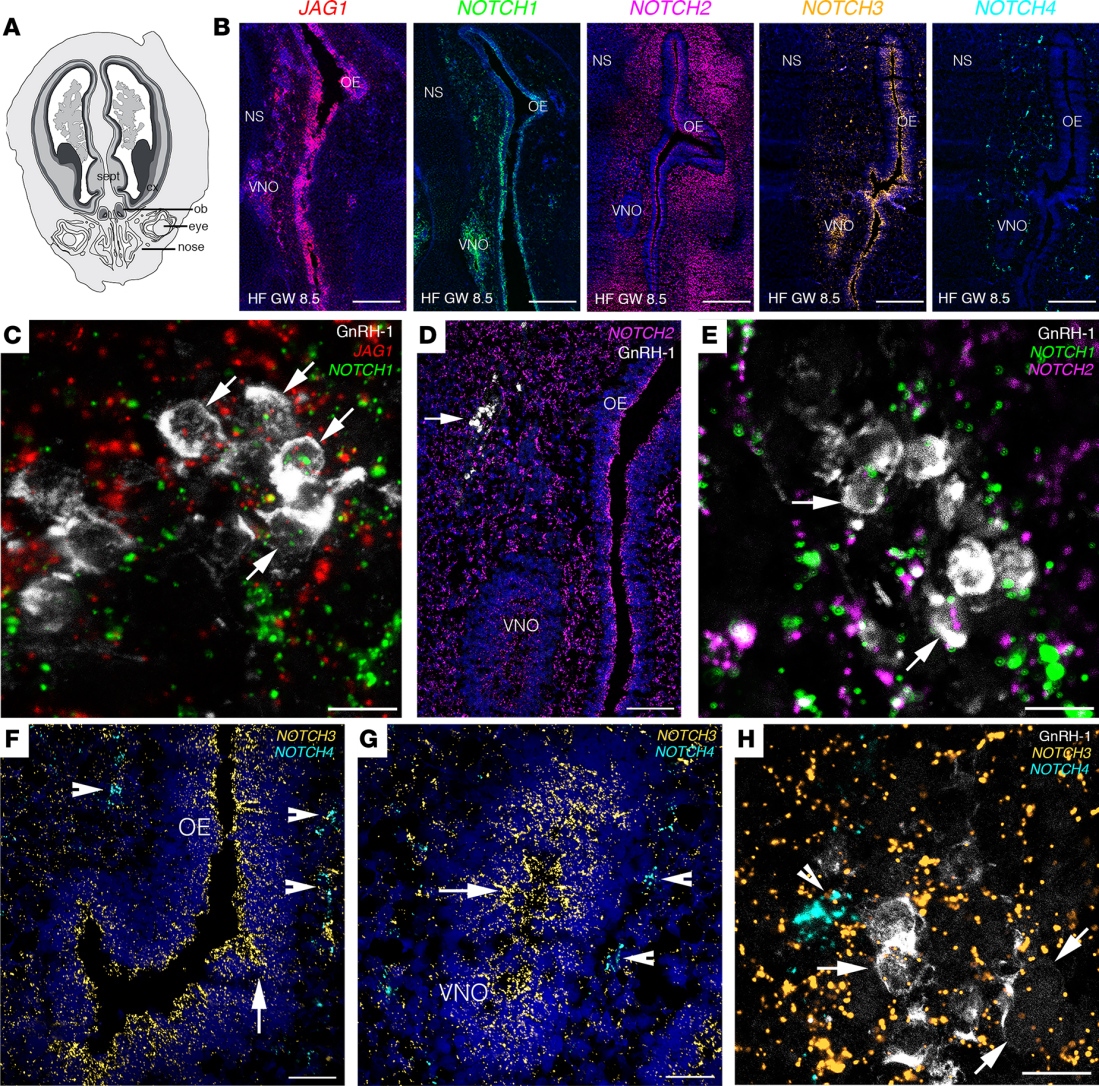
JAG1 and Notch receptors are expressed in GnRH cells and in the olfactory/vomeronasal systems of human fetuses. (**A**) Schematic representation of a GW 8.5 human fetus head (coronal view). (**B**) Expression of *JAG1* and *NOTCH1*-*NOTCH4* in coronal section of the nasal compartment of a GW 8.5 human fetus. (**C**–**H**) High-power micrographs of FISH analysis for *JAG1* and *NOTCH1–NOTCH4* coupled with immunofluorescence for GnRH. Arrows in **C** point to GnRH-1 neurons expressing *JAG1* and *NOTCH1* transcripts. Arrows in **D** point to GnRH-1 neurons. Arrows in **E** point to GnRH-1 neurons expressing *NOTCH1* and *NOTCH2* transcripts. Arrowheads in **F** and **G** point to *NOTCH4*-expressing cells, and arrows highlight *NOTCH3*-expressing territories in the OE and VNO. Arrows in **H** point to GnRH-1 neurons expressing *NOTCH3* transcripts and arrowhead depicts *NOTCH4*-expressing cells. OB, olfactory bulb; cx, cortex; sept, septum; OE, olfactory epithelium; VNO, vomeronasal organ; NS, nasal septum, HF, human fetus; GW, gestational weeks. Scale bars: 800 μm, **B**; 10 μm, **C**, **E**, and **H**; 200 μm, **D**; 100 μm, **F** and **G**. FISH experiments were replicated 3 times with similar results in *n* = 2 samples (GW 8.5 and GW 11).

Despite the presence of data regarding the expression of Notch signaling factors in the olfactory system of rodents (29), the expression of JAG1 signaling pathway in the developing olfactory, vomeronasal, and GnRH systems remains unknown. To fill this gap in knowledge, we investigated the expression of JAG1 and Notch paralogs’ transcripts (*NOTCH1*, *NOTCH2*, *NOTCH3*, *NOTCH4*), using multiplex fluorescence in situ hybridization (FISH), along the GnRH migratory route of human fetuses (Figure 1).

Here, we report the expression of *JAG1*, *NOTCH1*, and *NOTCH3* transcripts in the vomeronasal organ (VNO), in the developing olfactory epithelium (OE), as well as along chains of cells migrating across the nasal mesenchyme (Figure 1, A and B) in a fetus at GW 8.5. *NOTCH2* and *NOTCH4* expression was limited to the nasal tissue surrounding the VNO and OE (Figure 1B).

To assess the identity of cells expressing *JAG1* and the Notch paralogs, we coupled FISH assay for *JAG1* and Notch receptors with immunofluorescence for GnRH. We documented expression of *JAG1* (Figure 1C), *NOTCH1* (Figure 1, C and E), *NOTCH2* (Figure 1, D and E), and *NOTCH3* (Figure 1, F–H) transcripts in migratory GnRH neurons, whereas *NOTCH4* was detectable in the nasal mesenchyme but not in GnRH neurons (Figure 1, F–H).

We next performed multiplex FISH experiments to assess *JAG1* and *NOTCH1–4* expression patterns in olfactory ensheathing cells (OECs), an important component of the nasal migratory mass (MM) (4), which contributes to the correct development of GnRH neurons (30). At GW 11, we detected large clusters of OECs migrating across the nasal region (Supplemental Figure 1; supplemental material available online with this article; https://doi.org/10.1172/jci.insight.161998DS1) and expressing the OEC markers: low affinity nerve growth factor receptor (P75/NGFR), *NGFR*, and *S100β*. Our experiments showed that *Jag1* and *Notch1–3* transcripts were expressed in the OECs of the nasal compartment of human fetuses (Supplemental Figure 1, A–T), whereas *NOTCH4* was expressed at low levels in the nasal mesenchyme but not in OECs (Supplemental Figure 1, U–Y).

Together, these data illustrate a strong expression of *JAG1* and Notch receptors in GnRH neurons and in other cell types of the MM during early human fetal development.

### JAG1 and DLL1 are expressed along the GnRH migratory route in human fetuses.

**Figure 2:**
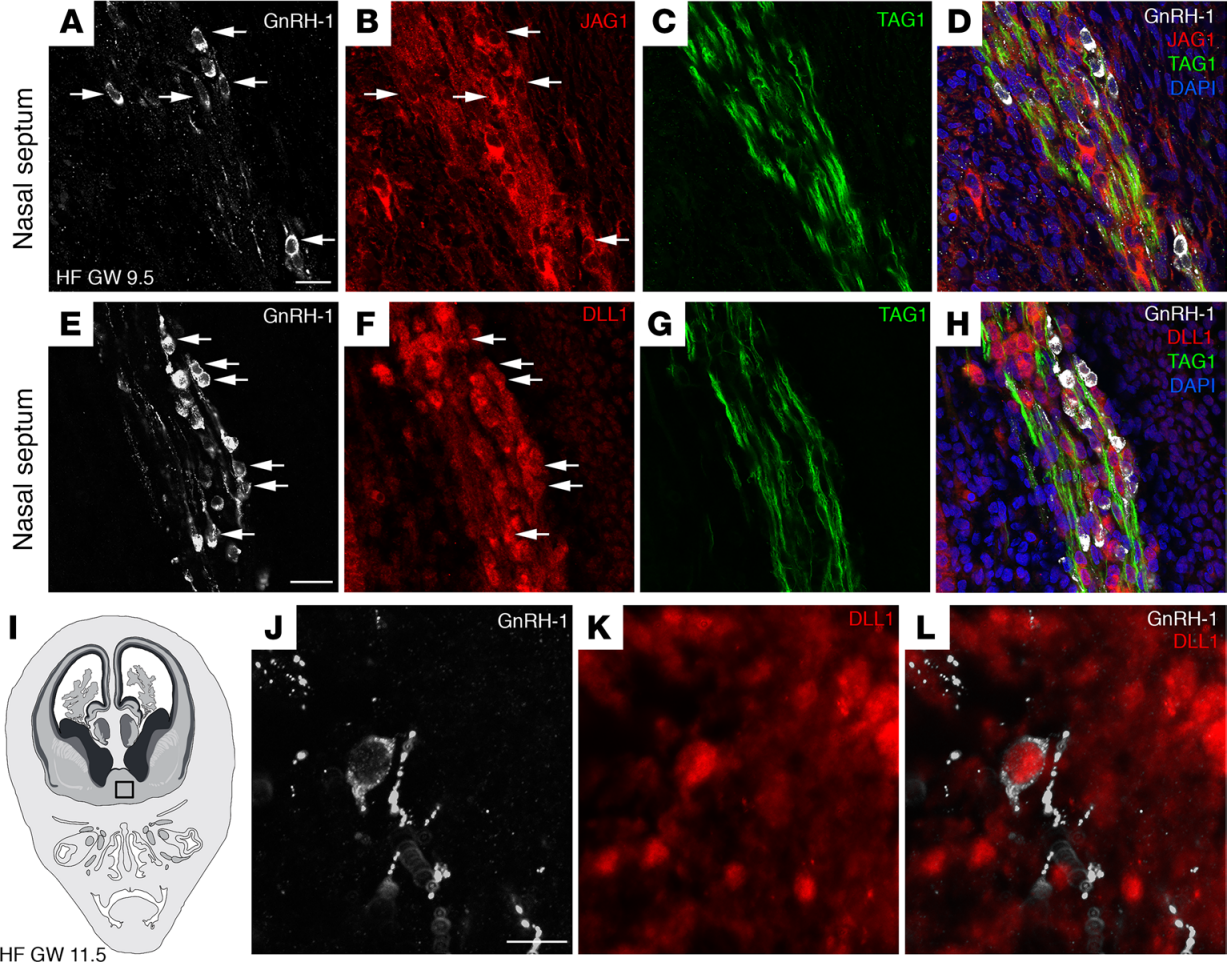
JAG1 and DLL1 proteins are expressed in GnRH neurons and in the olfactory/vomeronasal system of human fetuses. (**A**–**D**) Representative confocal images of the nasal septum of a GW 9.5 fetus immunostained for GnRH1 (white), JAG1 (red), and TAG1 (green). JAG1 (arrows, **B**) is expressed by GnRH1-expressing neurons (arrows, **A**), as well as by other neurons belonging to the MM and by vomeronasal/terminal fibers expressing TAG1. (**E**–**H**) GnRH1 (white), DLL1 (red), and TAG1 (green) expression in a coronal section of the nasal region of a GW 9.5 fetus. DLL1 is expressed by GnRH neurons (arrows, **E** and **F**), by other neurons belonging to the MM, and by vomeronasal/terminal fibers. (**I**) Schematic representation of a GW 11.5 human fetus head (coronal view). The black box indicates the forebrain region of immunofluorescence analysis displayed in **J**–**L**. (**J**–**L**) DLL1 is expressed by a GnRH neuron that entered the forebrain. Scale bars: 20 μm. The experiments were replicated 3 times with similar results in *n* = 2 samples (GW 9.5 and GW 11.5). HF, human fetus.

We further explored whether JAG1 protein is also expressed in migratory GnRH neurons. Coronal sections of a GW 9.5 fetus were immunostained for GnRH, JAG1, and transient axonal glycoprotein (TAG1), with the latter being a marker of the VNN/TN scaffold used by GnRH migratory neurons (4). Consistent with our FISH experiments, a robust expression of JAG1 was visible in GnRH neurons that migrated across the nasal septum (Figure 2, A–D). The expression of JAG1 was not restricted to GnRH cells, and it extended to other cell types that coalesce with GnRH neurons and along the olfactory/vomeronasal nerve fibers (Figure 2, A–D).

Given the lack of specific human anti–NOTCH1–4 antibodies that can be used for IHC detection, we next performed immunofluorescence experiments taking advantage of an antibody against the human homolog of the Notch Delta ligand 1, DLL1 (31), which has been previously validated to mirror the expression of NOTCH1 (32).

Here, we document expression of DLL1 in cells belonging to the MM, including GnRH cells (Figure 2, E–H). Additionally, the expression of DLL1 was also observed in GnRH cells that entered the forebrain at GW 11.5 (Figure 2, I–L).

Together, these data uncovered the expression of JAG1 and Notch Delta ligand 1 in human migratory GnRH neurons and along the VNN/TN scaffold, suggesting a role of JAG1 signaling in GnRH neuronal development.

### Jag1a, jag1b, and notch1a are expressed in the olfactory placode of zebrafish embryos.

Jag1-null mice have been reported to die from hemorrhage early during embryogenesis, exhibiting defects in the embryonic and yolk sac vasculature (33). Hence, they cannot be used to assess the potential role of Jag1 signaling in the development of GnRH neurons. To overcome this limitation, we took advantage of zebrafish as an in vivo model to investigate the expression pattern and functional role of jag1/notch signaling in the development of GnRH neurons.

Due to the genome duplication, zebrafish has 2 *JAG1* orthologs, *jag1a* and *jag1b* (34). Moreover, 2 forms of GnRH neurons were identified in zebrafish: GnRH2 and GnRH3 (35). The TN GnRH3 neurons are considered homologous to the mammalian GnRH1 neurons and, similar to mammals, they originate in the nasal olfactory placode (36, 37), they migrate from the nose to the brain along olfactory axons (38), and they display hypophysiotropic projections (39).

**Figure 3:**
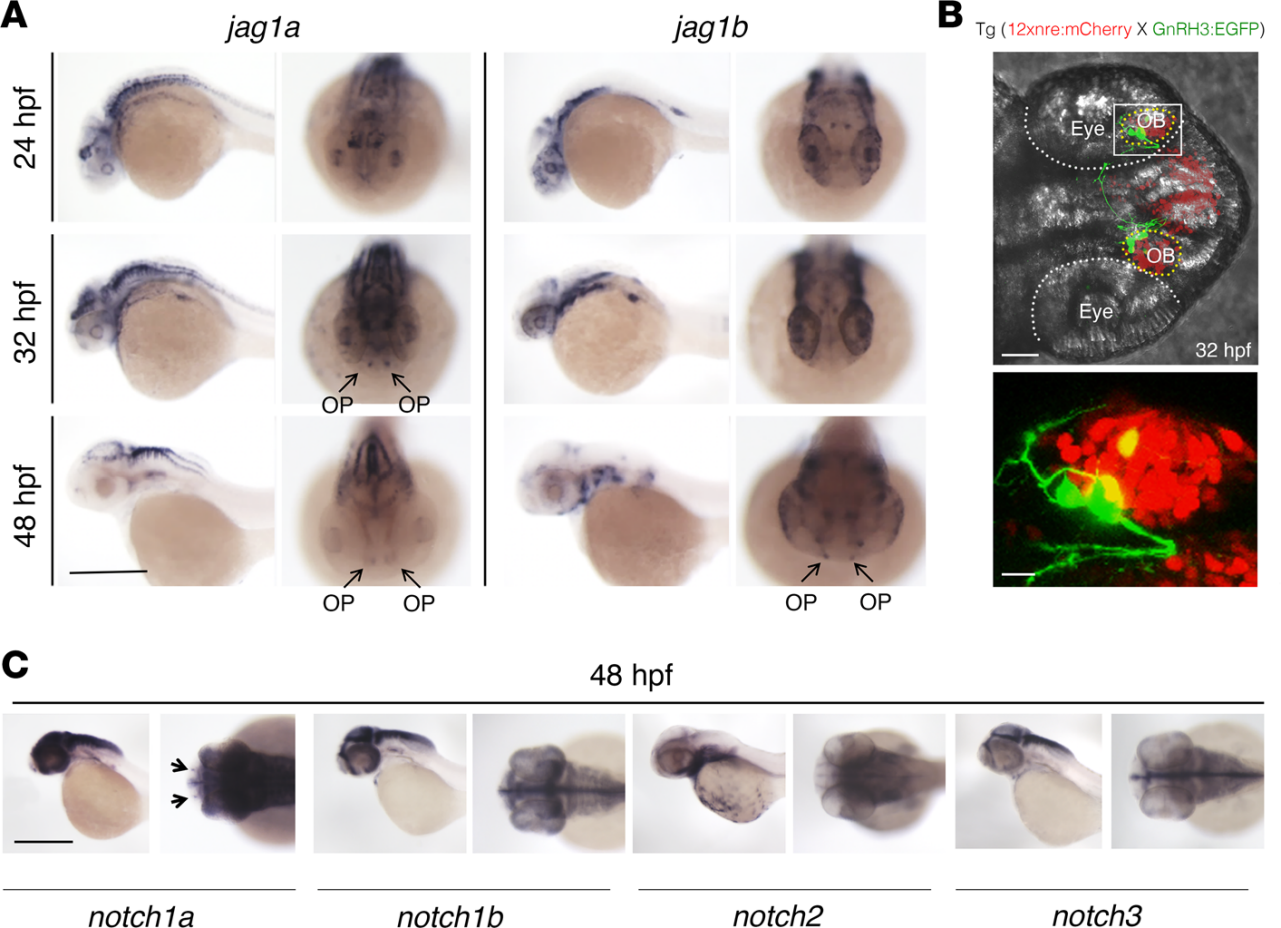
Jag1a, jag1b, notch1a, and gnrh3 mRNAs are expressed in the OP of zebrafish embryos at 48 hpf. (**A**) WISH on zebrafish embryos at 24 hpf, 32 hpf, and 48 hpf for *jag1a* and *jag1b* mRNAs. WISH was replicated 4 times with *n* = 15 WT embryos. *Jag1a* and *jag1b* are both expressed within the OP (arrows). (**B**) Representative confocal image of the double reporter line tg(12xnre:mCherry × GnRH3:EGFP) showing the expression of GnRH3 (green) and of the NRE (red) in the OBs of zebrafish embryos at 32 hpf. The experiments were replicated 3 times with *n* = 10 EGFP and mCherry double-positive embryos. (**C**) WISH on zebrafish embryos at 48 hpf showing *notch1a* mRNA localization in the OP (arrows). WISH was replicated 4 times with *n* = 15 WT embryos. Scale bars: 0.5 mm, **A** and **C**; 40 μm, **B** upper panel; 10 μm, **B** lower panel.

Using whole-mount in situ hybridization (WISH), we observed the expression of both transcripts at the level of the olfactory placode (OP) of zebrafish embryos in similar, but not identical, patterns (Figure 3A). *Jag1a* was detectable in the OP at 32 and 48 hours post fertilization (hpf), whereas *jag1b* appeared to start to be expressed in the OP at 48 hpf (Figure 3A). Furthermore, we analyzed the double reporter line, tg(GnRH3:EGFP × 12xnre:mCherry) (38, 40), which expresses the fluorescent proteins mCherry and the GFP under the control of the notch responsive element (NRE) and GnRH3 promoter, respectively. At 32 hpf, GnRH3 migratory neurons expressed the NRE at the level of OBs (Figure 3B), suggesting that GnRH3 neurons express notch receptors. WISH experiments for *notch1a*, *notch1b*, *notch2*, and *notch3* transcripts in embryos at 48 hpf verified that *notch1a* was expressed in the OP area (Figure 3C).

### Pharmacological inhibition of notch signaling alters the development of the GnRH3 system in zebrafish embryos.

The impact of Jag1/Notch signaling on GnRH3 neuronal development was further assessed by treating the tg(GnRH3:EGFP) embryos with the γ-secretase inhibitor DAPT, a well-known Notch inhibitor (41). The dose of 100 μM DAPT was selected as able to efficiently inhibit the Notch signaling in tg(12xNRE:mCherry) embryos without causing gross morphological alterations in the brain, in agreement with previous studies (40, 41) (Supplemental Figure 2, A–D).

**Figure 4:**
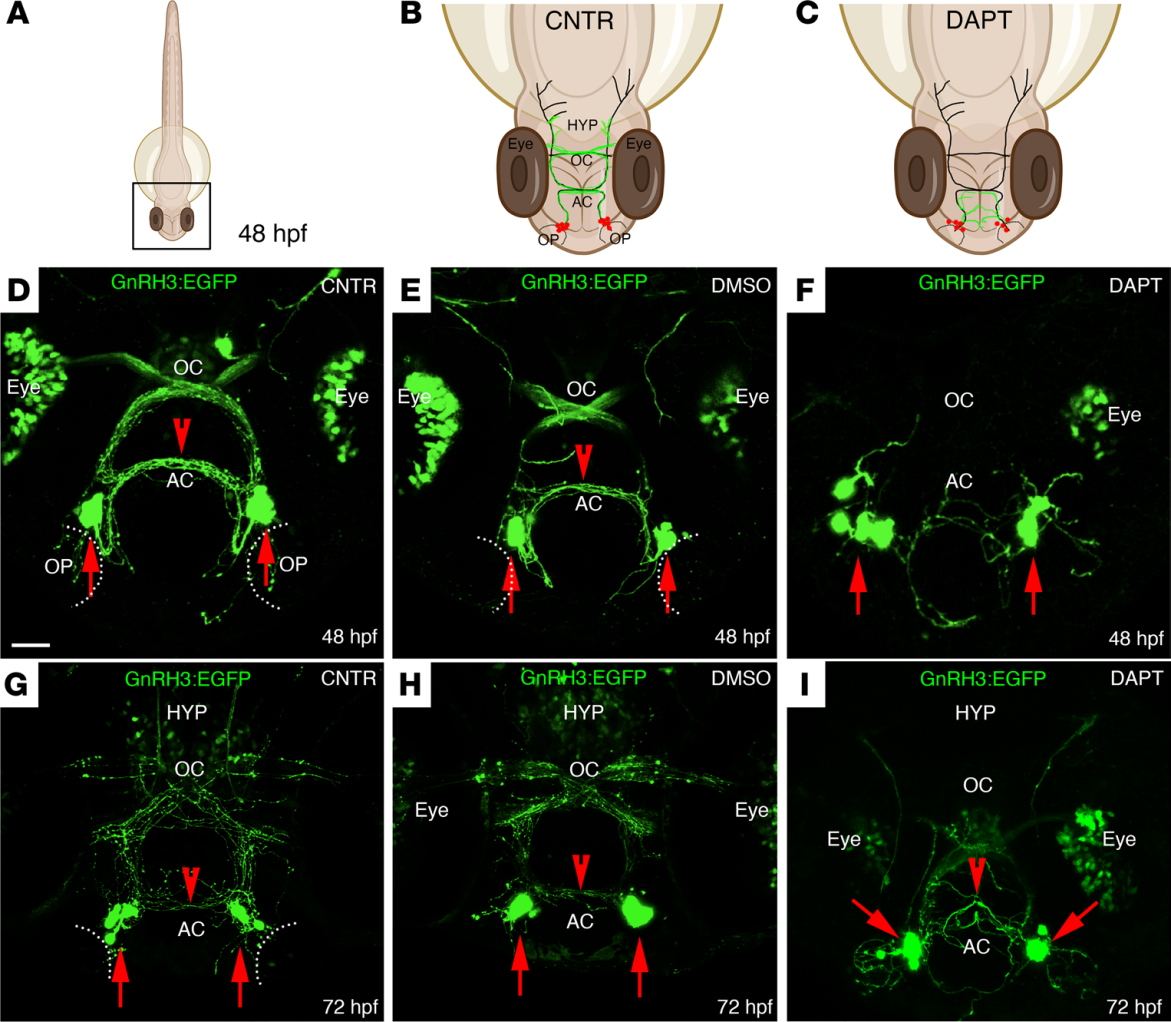
DAPT treatment affects the localization of GnRH3 neurons and their axonal projections. (**A**–**C**) Schematic representations of a 48 hpf zebrafish embryo. (**B** and **C**) Schematic representations of the boxed square area depicted in **A**, representing the distribution of GnRH3 cell bodies (red dots) and of GnRH3 neurites (green lines) in control (CNTR) and DAPT-treated embryos. The treatment was performed 3 times with *n* = 30 WT embryos/condition. (**D**–**F**) Representative confocal images of tg(GnRH3:EGFP) CNTR embryos (**D**), DMSO-treated embryos (**E**), and DAPT-treated embryos (**F**) at 48 hpf. (**G**–**I**) Representative confocal images of tg(GnRH3:EGFP) embryos at 72 hpf, under the different treatment conditions. Scale bar: 100 μm. AC, anterior commissure; OC, optic chiasm; HYP, hypothalamus.

Analysis of tg(GnRH3:EGFP) embryos at 48 hpf (Figure 4, A–C) treated from 6 hpf with DAPT pointed to a disorganization of the GnRH3 somata in the nasal compartment, a profound defasciculation of the GnRH3-EGFP fibers in the anterior commissure (AC), and lack of GnRH3 fibers’ innervation in the optic chiasm (OC) (Figure 4, D–F), compared with the control vehicle (1% DMSO). The defects observed in DAPT-treated embryos at 48 hpf are still present at 72 hpf, with embryos displaying a strong disorganization of the GnRH3 neuronal distribution in the nasal region, together with a pronounced defasciculation of the GnRH3 fibers in AC and OC, and absence of GnRH3 fiber projections across the hypothalamus (Figure 4, G–I).

These data suggest an involvement of Jag1/Notch signaling in the proper development and fiber projections of GnRH3 neurons.

### Jag1b knockdown affects the development of GnRH3 neurons in zebrafish.

Given the expression of *jag1a* and *jag1b* in the OP of zebrafish embryos, we then analyzed their potential involvement in GnRH3 development by knocking down the expression of *jag1* mRNAs by morpholino microinjections.

**Figure 5:**
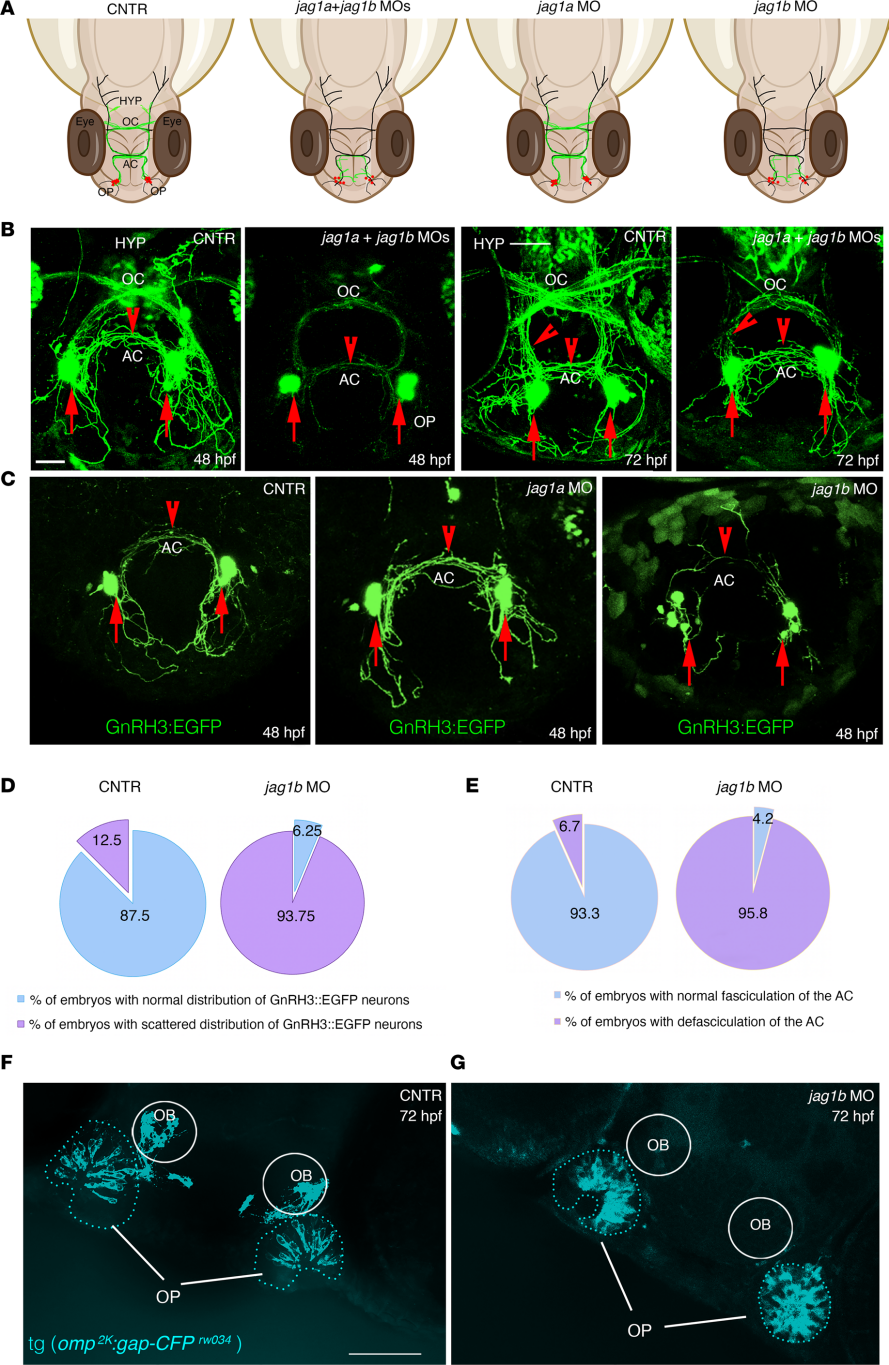
*jag1b*, but not *jag1a*, affects the distribution of GnRH3 neurons and their axonal projections. (**A**) Schematic representations of 48 hpf zebrafish embryos and representing the distribution of GnRH3 cell bodies (red dots) and of GnRH3 neurites (green lines) under the indicated experimental conditions. (**B**) Confocal analysis of tg(GnRH3:EGFP) control embryos and jag1a + jag1b double morphants at 48 hpf and 72 hpf, respectively. The experiments were performed 5 times with *n* = 15 EGFP-positive embryos/condition. (**C**) Confocal analysis of tg(GnRH3:EGFP) control embryos and jag1a and jag1b morphants at 48 hpf. Arrows indicate GnRH3-positive cells in the OP; arrowheads indicate GnRH3 fibers in the AC and innervating hypothalamus. HYP, hypothalamus. Red arrows indicate the somata of GnRH3^+^ cells. Red arrowheads highlight the GnRH3^+^ fiber in the AC or innervating hypothalamus at 72 hpf. (**D**) Percentage of embryos showing a scattered GnRH3^+^ cell phenotype between controls and jag1b morphants at 48 hpf. (**E**) Percentage of embryos showing a defasciculation of the AC between controls and jag1b morphants at 48 hpf. Mann-Whitney *U* test followed by a Fisher’s exact test. (**F** and **G**) Representative confocal images of tg(*omp^2k^:gap-CFP ^rw034^*) control (**F**) and *jag1b* MO embryos (**G**). The experiments were performed 4 times with *n* = 15 tg(*omp^2k^:gap-CFP ^rw034^*) embryos/condition. Scale bars: 100 μm, **B** and **C**; 50 μm, **F** and **G**.

The development and organization of the GnRH3 neuronal system were evaluated by confocal analysis of live tg(GnRH3:EGFP) embryos at 48 and 72 hpf (Figure 5A), according to the temporal development of GnRH3 neurons (42). In control animals at 48 hpf, GnRH3-positive cells were detectable in proximity to the OP and where branches of GnRH3 axons depart from the perikarya and project to the AC and OC (Figure 5B). Double *jag1a/jag1b* morphants showed profound alterations in the GnRH3 architecture. The GnRH3 fibers appeared disorganized and defasciculated, especially at the level of the AC and OC. At 72 hpf, the double morphants also presented a reduction of GnRH3 fibers that innervate the hypothalamus, as compared with the control embryos (Figure 5B).

Subsequently, to test the relative contribution of *jag1* orthologs on GnRH3 development, we generated single *jag1a*-knockdown (*jag1a*-KD) or *jag1b*-KD embryos.

In *jag1b* morphants at 48 hpf, the GnRH3 somata located in the nasal regions were highly disorganized, and the GnRH3-positive fibers projecting to the AC were completely defasciculated, whereas no defects of GnRH3 architecture were detectable in *jag1a* morphants, which appeared similar to those of controls (Figure 5C). Quantitative analysis revealed that in *jag1b* morphants, more GnRH3 displayed a scattered distribution within these regions as compared with control animals (Figure 5D) and an increased defasciculation of the GnRH3-positive AC fiber network (Figure 5E).

We then extended the analysis to the olfactory pathway because the ontogenesis and early development of GnRH3 neurons are closely associated with the development of the OP and because the olfactory development and GnRH ontogenesis are highly conserved among vertebrate species (43–45).

We took advantage of the tg(*omp^2k^:gap-CFP^rw034^*) reporter line, in which the cyan fluorescent protein (CFP) expression is under the control of the olfactory receptor marker (*omp*) promoter, a known marker of the olfactory axons (46–48).

In control embryos at 48 hpf, *omp* could be detected at the levels of olfactory neurons lying within the basal portion of the OP as well as the olfactory axonal projections to the OB (Figure 5F). Interestingly, *jag1b* morphants showed a disorganization of olfactory neurons in the OP and lacked OB projections (Figure 5G).

Since Notch signaling is known to properly shape embryonic development in several species, including zebrafish (49–54), we next evaluated whether impairment of jag1b signaling affects zebrafish development. The analysis of embryonic morphology at 24 and 48 hpf showed that the majority of in *jag1b* morphants developed normally, except for a pericardial edema detectable at 48 hpf. Other defects, including the enlargement and blood stasis at the level of the caudal vein plexus and cerebral edema were detectable in 15%–37% of morphants (Supplemental Figure 3A). To further exclude the presence of embryonic delay after *jag1b* morpholino injection, the expression of *hoxA* genes was analyzed by quantitative PCR (qPCR) (48, 55). This analysis revealed no differences in the expression levels of *hoxA7a* and *hoxA10b* between controls and morphants at both 24 and 48 hpf (Supplemental Figure 3, B and C). Finally, WISH experiments revealed comparable expression of markers of forebrain determination, *islet1*, *neurog1*, and *shha*, between control and *jag1b* morphants (Supplemental Figure 3, D and E), excluding that the defects observed on GnRH3 neurons were associated with embryonic delay or gross morphological alterations of the brain.

Overall, our data identified a potentially novel role for *jag1b* signaling in the proper development of GnRH3 neurons as well as in the correct targeting of GnRH3 and olfactory fibers.

### Notch inhibition impairs GnRH cell motility in vitro.

**Figure 6:**
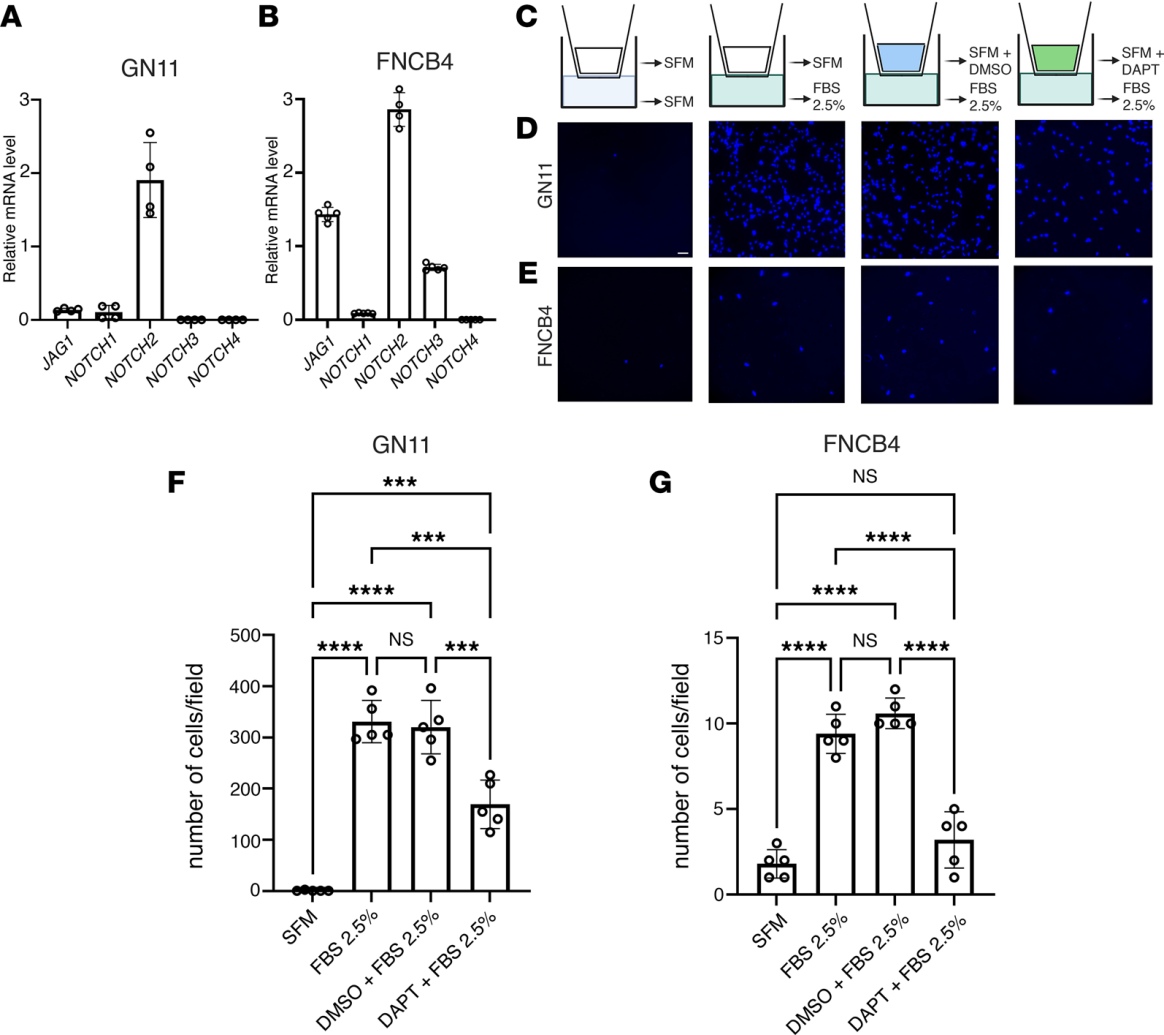
Notch inhibition impairs GnRH cell motility in vitro. (**A** and **B**) qRT-PCR analysis of indicated transcripts normalized to *Actb* in GN11 cells (*n* = 4) and to *S18* in FNCB4 cells (*n* = 5 for all genes, *n* = 4 for *NOTCH2*). (**C**) Schematic depiction of the Transwell assays. (**D** and **E**) Representative photomicrographs showing Hoechst nuclear staining of the migrated GN11 (**D**) and FNCB4 cells (**E**) after the different treatment conditions indicated in **C**. (**F** and **G**) Bar graphs illustrate the mean number of migrated GN11 cells (**F**) and FNCB4 cells (**G**) (*n* = 5 per condition). Comparison between the different treatment conditions were performed using a 2-way ANOVA followed by Tukey’s multiple-comparison post hoc test; ***P < 0.0005 and ****P < 0.0001. Data are represented as the mean ± SEM. Scale bar: 50 μm.

Manipulation of the GnRH migratory system and functional investigation on these neurons have been challenging because of their limited number (800 in rodents) and anatomical dispersal along their migratory route (9). The generation of immortalized GnRH neuronal cell lines has allowed the study of immature migratory (GN11 cells) GnRH neurons (56). To assess whether immortalized GnRH cell lines retain expression of Jag1 and Notch receptors, we performed quantitative real-time PCR (qRT-PCR) analysis (Figure 6A). Our data show that GN11 cells expressed *Jag1*, *Notch1*, and *Notch2* transcripts (Figure 6A). We also performed similar experiments taking advantage of the human GnRH-secreting neuroblast line FNCB4 (27, 28), a primary long-term cell culture previously established, cloned, and propagated in vitro from human fetal OE (27). These cells have been shown to preserve cell motility in vitro (57).

Consistent with the transcripts’ expression pattern described in human fetal sections (Figure 1, C–E and H), our in vitro experiments revealed that FNCB4 cells expressed *JAG1* and *NOTCH1-NOTCH3* but not *NOTCH4* mRNAs (Figure 6B).

Since both GN11 and FNCB4 cells expressed *Jag1* and *Notch* receptors, we next aimed at assessing the impact of the inhibition of the endogenous Jag1/Notch signaling on GnRH cell motility, using DAPT at the same concentration (100 μM) as in our in vivo experiments. To this purpose, we used GN11 and FNCB4 cells in a Transwell plate assay (Figure 6C). We seeded GN11 and FNCB4 cells in the upper chamber in the presence of serum-free medium (SFM) and added FBS 2.5% as a positive stimulus for cell motility in the lower compartment of a Transwell plate. We showed that FBS 2.5% induced a significant migration of both cells, though GN11 cells displayed a higher degree of motility as compared with FNCB4 cells (Figure 6, D–G). The FBS-dependent induction of cell motility of both cell types was prevented by the pharmacological antagonist of Notch pathway plated in the upper chamber (Figure 6, D–G).

In combination with the in vivo data, these results suggest that Jag1/Notch signaling stimulates GnRH cell motility in zebrafish, mice, and humans.

### Individuals with CHH harbor JAG1 rare variants.

**Figure 7:**
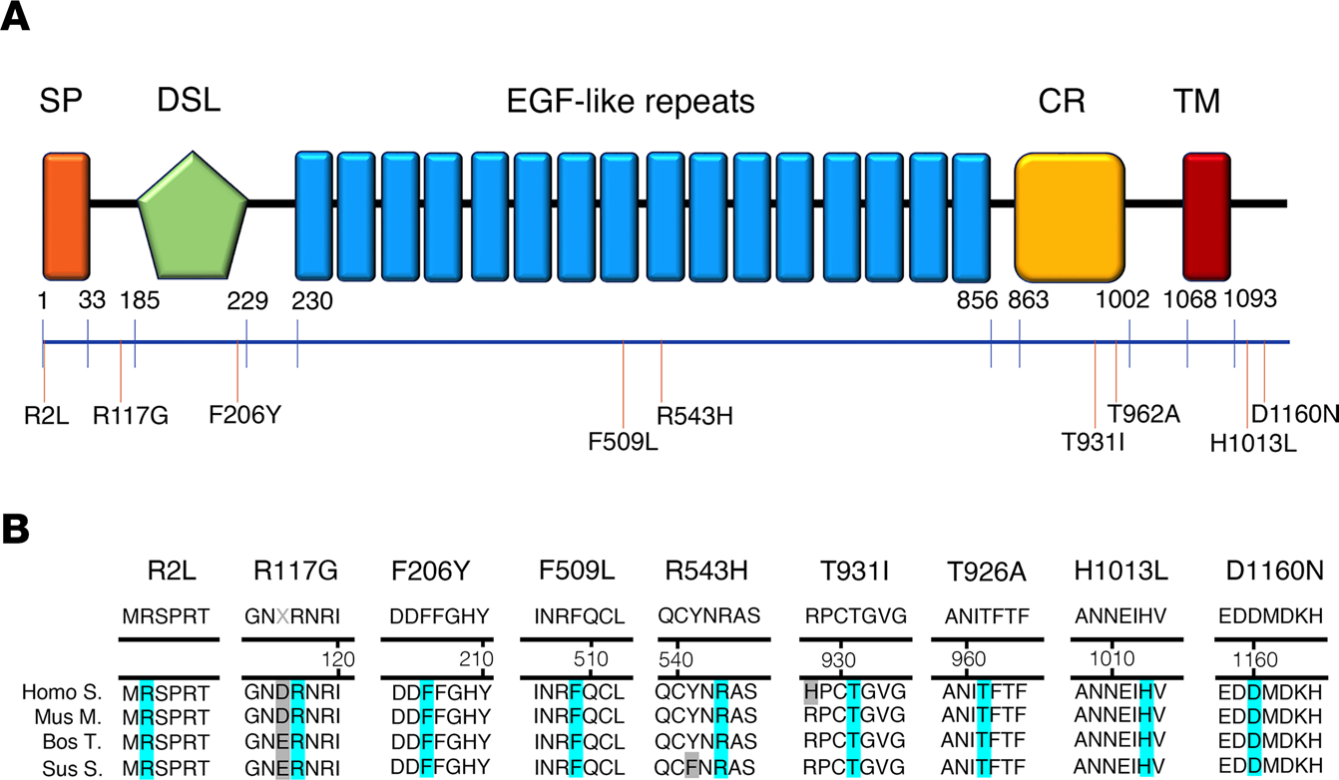
JAG1 heterozygous mutations in CHH probands. (**A**) Schematic illustration of JAG1 mutations in nCHH and KS probands. The signal peptide (SP), the Delta:Serrate:LAG-2 (DSL) domain, the EGF-like repeats, the cysteine-rich region (CR), and the transmembrane domain (TM) are represented. The relative position of the variants is indicated on the illustration. (**B**) Alignment of partial protein sequences of JAG1 orthologs showing in blue the amino acid residues evolutionarily conserved.

In this study, we performed target next-generation sequencing in 51 KS and 76 normosmic CHH (nCHH) probands (Milan cohort) and identified 4 heterozygous putative pathogenic rare sequence variants (RSVs) in *JAG1* among 4 CHH probands: p.Arg117Gly, p.Phe206Tyr, p.Thr931Ile, and p.Asp1160Asn. Further, within the Lausanne cohort, 340 CHH probands (144 nCHH and 196 KS) underwent next-generation whole-exome sequencing, and 5 heterozygous variants were identified: p.Arg2Leu, p.Asn504Ser, p.Phe509Leu, p.Arg543His, p.Thr962Ala, and p.His1013Leu (Figure 7, A and B, and Tables 1 and 2).

None of these variants were predicted to affect splicing. The p.Arg2Leu and the p.Arg117Gly variants lie in the N-terminal protein domain; p.Phe206Tyr in the DSL domain; p.Phe509Leu and p.Arg543His in the EGF-like repeats; p.Thr931Ile and p.Thr962Ala in the cysteine-rich region; p.His1013Leu lies within regions; whereas p.Asp1160Asn in the transmembrane domain. All affected amino acids were highly conserved across species (Figure 7B).

We observed prepubertal onset in 8 out of 9 probands carrying *JAG1* mutations. One proband had nCHH with no other major associated nonreproductive phenotypes (Table 1).

### JAG1 mutations result in altered expression and/or intracellular retention in vitro.

**Figure 8:**
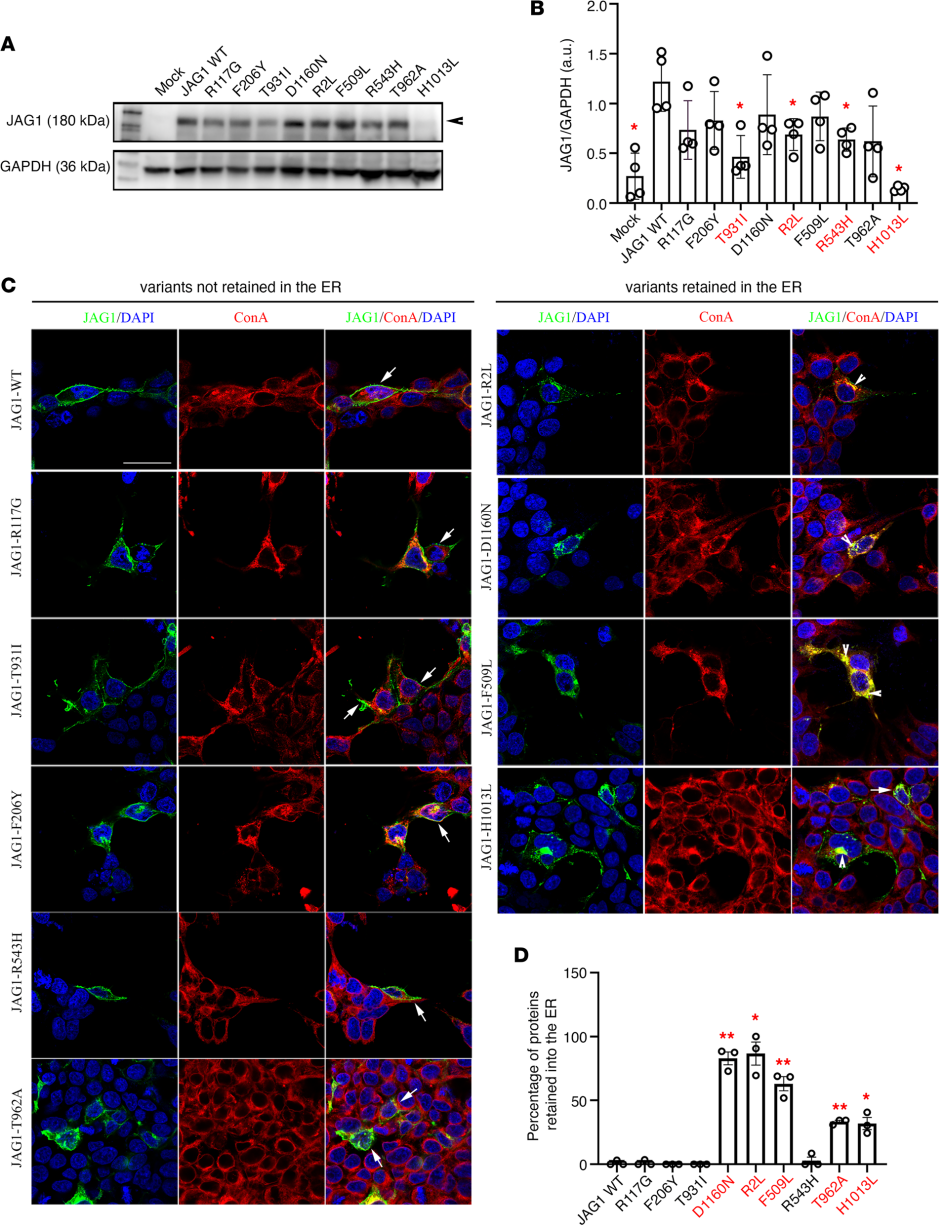
Functional validation of JAG1 variants in vitro. (**A**) Representative Western blot gels showing JAG1 and GAPDH expression in cell lysates of HEK293T cells transiently transfected with the empty vector (mock), with the JAG1 WT vector, or with each of the JAG1 variants. (**B**) Bar graph illustrates the mean ratio ± SEM JAG1 over GAPDH (*n* = 4 for all). Mann-Whitney *U* test followed by a Fisher’s exact test, *P < 0.05. a.u., arbitrary unit. (**C**) Confocal representative images of HEK293T cells transfected with JAG1 WT and JAG1 mutants and immunostained for JAG1 (green) and ConA (red). Nuclei were counterstained with DAPI (blue). Arrows point to the membrane JAG1 localization and arrowheads point to intracellular JAG1 retention. The experiments have been replicated 3 times. (**D**) Graph showing the percentage of JAG1 retention in the ER. Comparisons between treatment groups (*n* = 3 cultures/condition and *n* = 100 total cells analyzed/condition) were performed using a 1-way ANOVA followed by Tukey’s multiple-comparison post hoc test; *P < 0.05, **P < 0.005. Data represent mean ± SEM. Scale bar: 20 μm.

We transiently transfected HEK293T cells with WT and mutant tagged JAG1 cDNAs for ectopic JAG1 protein synthesis. Western blot analysis of WT-transfected HEK293T cells showed that JAG1 protein was detected in the cell extract (Figure 8A). Among the different mutants, we found 4 variants (T931I, R2L, R543H, H1013L) with significantly reduced expression as compared with the reference JAG1-WT–transfected cells (Figure 8, A and B), suggesting impaired protein synthesis or rapid degradation.

We next performed immunofluorescence assays to investigate the cellular localization of the JAG1 variants transfected in HEK293T cells. We combined an antibody directed against the human JAG1 with an antibody directed against Concanavalin A (ConA), which is a marker of ER (Figure 8C). These experiments showed that among the 9 variants, 5 were retained in the ER (T962A, R2L, D1160N, F509L, and H1013L; Figure 8, C and D). Interestingly, 2 mutants (R2L and H1013L) displayed both altered protein expression and cytoplasmic retention in HEK293T transfected cells.

Taken together, these in vitro results validate 7 out of the 9 JAG1 mutants as causing an impairment of protein synthesis and subcellular localization.

## Discussion

In rodents and humans, early GnRH neurons migrate together with a heterogeneous coalescence of placode-derived and neural crest–derived migratory cells and olfactory axons, collectively called the MM (4, 58–60). Among these cells, OECs, a type of glial cell with myelinating capacity, represent an important population of cells enwrapping the VNN/TN and GnRH neurons (4, 30) as previous studies showed that defects in OECs’ development result in an accumulation of GnRH cells in the nasal region (30).

The expression of Notch1/Jag1 in the developing OE and in OECs has been previously reported in several species, including mouse and chicken (12, 61). Interestingly, a function for Notch signaling in cell fate specification of olfactory and vomeronasal sensory neurons has been also demonstrated (13, 29).

In this study, we provide evidence for expression of JAG1 and Notch receptors during early human fetal development in migratory GnRH neurons as well as in the other cellular components of the MM, namely the OECs and the vomeronasal and olfactory axons. Based on these expression data and abovementioned murine studies, we speculate that Jag1/Notch signaling could play a role in the GnRH neuron development.

By combining WISH with pharmacological Notch inhibition and downregulation of the zebrafish *jag1b* gene (orthologous of JAG1) in the tg(GnRH3:EGFP) transgenic zebrafish strain, we revealed that *jag1b* is required for the proper organization and fasciculation of the neurites of GnRH3:EGFP neurons crossing the AC and projecting toward the hypothalamus. Moreover, genetic silencing of *jag1b* in the *omp^2k^:gap-CFP^rw034^* transgenic zebrafish strain led to defects in olfactory targeting from the OP to the OB (Figure 5, F and G). These results suggest a role for *jag1b* in the guidance and connectivity of olfactory axons and the organization of the GnRH3 axons.

In addition to the negative effects on GnRH3 development mediated by the genetic KD of *jag1b* in zebrafish, our pharmacological experiments, performed in vivo and in vitro, respectively, provide strong evidence for an autocrine and/or paracrine role of Jag1/Notch signaling in GnRH cell migration in zebrafish, mice, and humans. This is in agreement with previous studies providing evidence that migration of postmitotic neurons is regulated by Notch signaling (62).

In this study, we could not dissect the precise intracellular molecular mechanisms underlying the Jag1-mediated regulation of GnRH cell motility and olfactory axonal extension. Nevertheless, these may involve modifications of Notch intracellular trafficking and activity (63), mobilization of intracellular cytosolic Ca^2+^ concentration (64) that is also involved in regulation of GnRH3 cell motility and neuronal activity (37), or modification of expression of Notch target genes involved in cytoskeletal rearrangements.

Finally, Golan and coauthors have provided elegant evidence suggesting that connectivity and synchronized firing activity of GnRH3 neurons during the migrational pause are responsible for the maturation of GnRH3 neurons and transition from the nasal region into the forebrain (37). Here, we showed that downregulation of *jag1b* disrupted the GnRH3 clusters as shown by the disaggregation and dispersion of GnRH3 neurons in the nasal region as compared with control conditions. We can thus speculate that by losing their cluster-like organization, as detected in *jag1b* morphants, GnRH3 cells could also reduce their neuronal activity synchronization, which is mandatory for proper migration toward their final hypothalamic destination (37).

The impairment of development/migration of GnRH neurons is the main cause of KS, an inherited disorder characterized by hypogonadism and anosmia. Based on our human and zebrafish data, we searched for possible mutations in *JAG1* in 2 cohorts of patients with CHH/KS and identified 9 heterozygous putative pathogenic RSVs (R117G, F206Y, Y931I, D1160N, R2L, F509A, R543H, T962A, H1013L). We identified *JAG1* heterozygous variants in 1.9% of CHH probands (9 out of 467). This is consistent with the genetic feature of CHH in which the majority of known CHH genes have a low mutational prevalence (<5%) (6). Over the past few years, the traditional Mendelian view of CHH as a monogenic disorder has been revised following the identification of oligogenic forms of CHH (65). CHH is genetically heterogeneous, with both sporadic and familial cases, and several modes of inheritance have been identified, including X chromosome–linked recessive and autosomal recessive and dominant (6). To date, several genes have been implicated in KS and/or CHH etiopathology, which account for approximately 50% of cases (8), suggesting that other possible candidate genes are yet to be discovered. Consistent with the oligogenic basis of CHH, we identified 7 *JAG1* variants, which segregated with mutations in genes previously reported in patients with CHH/KS: *SEMA3A*, *SEMA3E*, *SEMA7A*, *IL17DR*, *PROKR2*, or *FGFR1* (66–68). In this study, we validated in vitro the *JAG1* gene allelic variants, and we showed that 7 out of the 9 *JAG1* gene variants impaired protein synthesis or localization. *JAG1* is well known to be involved in Alagille syndrome (ALGS); however, none of our participants presented any of the ALGS signs.

Haploinsufficiency caused by truncation or early transcriptional termination of *JAG1* account for more than 80% of mutations seen in ALGS patients whereas *NOTCH2* variants occur in less than 3% of patients (26). Consistent with the documented involvement of JAG1 in the pathogenesis of ALGS, the heterozygous JAG1 variations with a more severe or complete loss of function are associated with ALGS, whereas CHH phenotype is associated with 7 rare missense variants here characterized by a partial impairment of protein synthesis or localization. Future investigations would be required to assess whether the *JAG1* variants identified in CHH/KS and in ALGS patients may respectively disturb protein function in different and, perhaps, opposite ways (i.e., with some variants inhibiting and other mutations inducing a persistent activated state of the NOTCH receptor, JAG1). Both ALGS and CHH are diseases characterized by a variable expression and penetrance, consistent with the distinct phenotype documented in the relatives of the probands, which suggests that mechanisms, yet to be identified, are likely to contribute to the clinical expression of *JAG1* gene defects.

To our knowledge, this is the first report that provides experimental evidence for the association between Notch signaling defects and CHH. Very little is reported on fertility and age of puberty in patients with ALGS, and severe clinical symptoms affecting most ALGS patients may mask the clinical signs of a mild CHH. Hence, our genetic findings, together with our animal and cellular data, put forward the possibility that CHH may be a frequent but disregarded symptom of ALGS.

In conclusion, we provide compelling evidence that Jag1/Notch signaling has a role in the development of GnRH neurons/olfactory system, and we propose that Jag1/Notch insufficiency may contribute to the pathogenesis of CHH in humans.

## Methods

### Participants

The studied cohort consists of 467 unrelated CHH patients recruited since 2008. Anonymous patient data, at the time of diagnosis, before any therapy, were collected either prospectively or retrospectively and a clinical database was created. All participants were affected with CHH (age range at diagnosis: 13–70 years), including patients with normal olfaction (*n* = 220) or olfactory defects (hypo- or anosmia, *n* = 247) as demonstrated either by a smell test (Brief Smell Identification Test, Sensonics), University of Pennsylvania Smell Identification Test (Sensonics), or Sniffin’ Sticks Identification test (Burghart, Germany); MRI; or both. CHH was defined as (a) manifestations of hypogonadism associated with low testosterone and inappropriately low/normal gonadotropins and (b) absence of any known acquired CHH cause (i.e., expansive hypothalamic/pituitary lesions, hemochromatosis, etc.), or multiple pituitary hormone defects. To omit the functional hypothalamic defects, exclusion criteria were (a) severe weight loss (body mass index < 18.5 kg/m^2^) (69), (b) intensive exercise (>5 hours/week), and (c) chronic illness and psychiatric disorders.

### DNA sequencing and bioinformatic analyses

The 2 cohorts of patients with CHH were analyzed using 2 methods. In Milan, each patient underwent a genetic investigation, using a targeted next-generation sequencing technique, to search for rare allelic variants. The genomic DNA was extracted of each patient from peripheral blood lymphocytes using Gene Catcher gDNA 96 × 10 mL Automated Blood kit (Invitrogen). The CHH gene panel was designed using Illumina Design Studio and included the following CHH candidate genes: *ANOS1* (*KAL1*), *FGFR1*, *PROKR2*, *PROK2*, *GNRHR*, *GNRH1*, *GNRH2*, *KISS1*, *KISS1R*, *TAC3*, *TACR3*, *HS6ST1*, *FGF8*, *CHD7*, *DUSP6*, *FEZF1*, *FGF17*, *FLTR3*, *IL17*, *SEMA3A*, *SEMA3E*, *SEMA7A*, *SOX2*, *SOX10*, *SPRY4*, *WDR11*, *HESX1*, *NELF*. The 28 CHH genes consistently represented in all sequence capture panels were assessed for the purposes of this study. Libraries were prepared using Illumina Nextera Rapid Capture Custom Enrichment kits according to the manufacturer’s protocols. All regions not correctly sequenced were recovered with NexteraVR DNA Library Preparation Kit (Illumina).

In Lausanne, whole-exome sequencing was performed using several standard protocols, such as Integrated DNA Technologies and Twist Bioscience, and sequenced on the Illumina 4000 by BGI Genomics or Health 2030 Genome Center. Genotype calling and variant annotation were performed using the Genome Analysis Toolkit’s Best Practices (70) and an in-house bioinformatics pipeline as previously described (71).

For subsequent analyses, we included as “rare variants” (72) all known pathogenic, or rare nonsynonymous or splicing-site variants (MAF ≤ 0.01) and novel nonsynonymous or splicing-site variants. The frequency and the functional annotation of the identified variants were checked in public and licensed databases (Ensembl, UCSC Genome browser, 1000 Genome project, ExAC Browser, NCBI, HGMD professional, gnomAD), considering all ethnic groups (Lausanne cohort) and Europeans only (Milan cohort). As previously reported (71, 73) we excluded common nonsynonymous variants with MAF > 0.01, synonymous, intronic, and 5′ or 3′ UTR variants. Several protein-prediction algorithms, such as SIFT (74), Polyphen2 (75), MutationTaster2 (76), and CADD (77), were applied. Moreover, the ACMG guidelines were used to classify the pathogenicity of the variants (78). Each variant found was confirmed by Sanger direct sequencing using BigDyeVR Terminator v.3.1 Cycle Sequencing Kit (Life Technologies) on a 3100 DNA Analyzer from Applied Biosystems.

### Human fetuses

Nonpathological human fetuses were obtained at GW 8.5, GW 9.5, GW 11, and GW 11.5 from voluntarily terminated pregnancies after written informed consent of the parents (Gynaecology Department, Jeanne de Flandre Hospital, Lille, France) and they have been either fixed and frozen or fresh-frozen as detailed below. The fetuses were stored in the French Inserm biobank (HuDeCA).

### Multiplex FISH combined with immunofluorescence

Two human fetuses at GW 8.5 and 1 at GW 11 were snap-frozen in liquid nitrogen and stored at –80°C until use. Human tissues were cryosectioned using a CM3050 Leica cryostat at 16 μm.

FISH was performed on frozen sections of the nasal regions by RNAscope Multiplex Fluorescent Kit v2 according to the manufacturer’s protocol (Advanced Cell Diagnostics). Specific probes were used to detect *JAG1* (546181-C1), *NOTCH1* (311861-C2), *NOTCH2* (520481-C1), *NOTCH3* (558991-C1), *NOTCH4* (409631-C2), *S100B* (430891-C2), and *NGFR* (406331-C3) mRNAs. Hybridization with a probe against the *Bacillus subtilis* dihydrodipicolinate reductase (*dapB*) gene (320871) was used as a negative control and 3-Plex Positive (320861) as positive control. Immunofluorescence against GnRH was performed, after RNAscope staining, as previously reported (79). Briefly, the sections were rinsed with 0.1 M PBS and incubated at 4°C overnight with the previously validated (4) guinea pig anti-GnRH (a gift from Erik Hrabovszky, Laboratory of Endocrine Neurobiology, Institute of Experimental Medicine of the Hungarian Academy of Sciences, Budapest, Hungary) diluted at 1:1,000 in 0.1 M PBS containing 0.3% Triton X-100 and 10% normal donkey serum. The sections were then washed in PBS and incubated for 1 hour with Alexa Fluor 647 AffiniPure Donkey Anti–Guinea Pig IgG (Jackson ImmunoResearch, 706-605-148) diluted 1:400 in PBS and counterstained with DAPI nuclear staining (1:10,000; Thermo Fisher Scientific, 62248). Sections were mounted using Mowiol (MilliporeSigma, 475904) and analyzed using an LSM 710 confocal microscope (ZEISS).

### Immunofluorescence on human sections

Human tissues were cryosectioned (Leica cryostat) at 18 μm. Sections were thawed at room temperature and boiled at 80°C–90°C in the citrate buffer (9 mL citric acid buffer 0.1 M + 41 mL sodium citrate buffer 0.1 M + water to 1 L) for the antigen retrieval. Slides were washed 3 times in PBS 1× and incubated for 3 days in the primary antibody solution (PBS 1×, 0.3% Triton X-100, 2% normal donkey serum) at 4°C. the primary antibodies used were anti-DLL1 rabbit 1:100 (Abcam, ab10554), anti-Jag1 rabbit 1:100 (Cell Signaling Technology, 2155), anti-TAG1 goat 1:500 (R&D Systems, AF4439), and anti-GnRH guinea pig 1018, 1:10,000 (gift by Erik Hrabovszky, Laboratory of Endocrine Neurobiology, Institute of Experimental Medicine of the Hungarian Academy of Sciences, Budapest, Hungary). Slides were rinsed 3 times in PBS 1× and incubated in the secondary antibody solution (PBS 1×, 0.3% Triton X-100, 2% normal donkey serum) for 1 hour at room temperature. The secondary antibodies used were the Alexa Fluor 568 Donkey anti-Rabbit IgG 1:500 (Invitrogen, A10042), Alexa Fluor 488 Donkey anti-Goat IgG 1:500 (Invitrogen, 11055), and Alexa Fluor 647–conjugated AffiniPure Donkey Anti–Guinea Pig IgG 1:500 (Jackson Immuno Research, 706-605-148). After 3 additional washes, nuclei were counterstained with DAPI. Slides were mounted with Mowiol and pictures were taken using ZEISS LSM 710 AiryScan confocal microscope.

### Cell culture, transfection protocol, and Western blot analysis

HEK293T cells (sourced from ATCC) and GN11 cells (56) (gift of Sally Radovick, Rutgers University, New Brunswick, New Jersey, USA) were grown in monolayer at 37°C under 5% CO_2_ in DMEM (Thermo Fisher Scientific, 11965092) containing 100 μg/mL streptomycin, 100 U/mL penicillin (Thermo Fisher Scientific, 15140122), supplemented with 10% FBS (Thermo Fisher Scientific, 26140079). FNCB4 cells were previously established, cloned, and propagated in vitro from the human fetal OE (27) and characterized as migratory GnRH-secreting neuroblasts (28). Cells, cryogenically preserved, were cultured at 37°C in 5% CO_2_ atmosphere in Coon’s modified Ham F-12 medium (MilliporeSigma, F6636) supplemented with 10% FBS. Cells were maintained below full confluence by trypsinization and seeding onto 10 cm² dishes. HEK293T cells were seeded (2.5 × 10^5^ cell/well) on 6-well plates and transfected 24 hours later with Lipofectamine 3000 Reagent (Thermo Fisher Scientific, L3000015) according to the data sheet, using 1 μg of plasmid.

Western blot experiments were carried out on HEK293T cell lysates using antibodies anti-Jagged1 (28H8) (Cell Signaling Technology, 2620) and anti-Actin 1:5,000 (MilliporeSigma, A5060) as internal control.

### Western blot

We have extracted total protein extract using RIPA buffer in HEK293T cells, after transfection with the empty plasmid of JAG1 variants. Homogenates were centrifuged at 4,000*g* for 10 minutes at 4°C to remove cell debris. Protein content was assayed by the BCA protein assay kit (Thermo Fisher Scientific, 23225). Total proteins were fractionated by SDS electrophoresis on NuPage 4%–12% Bis-Tris gel (Thermo Fisher Scientific, NP0335BOX) and electrotransferred onto nitrocellulose membranes (Hybond-C super, Amersham Biosciences, 10600002). After blocking with TBS supplemented with 5% nonfat dry milk and 0.1% Tween 20, membranes were incubated overnight at 4°C with anti-JAG1 1:1,000 (28H8) rabbit, and anti-GAPDH 1:5,000 rabbit (MilliporeSigma, G9545) as internal control. After 3 washings in TBS with 0.1% Tween solution, membranes were incubated for 1 hour at room temperature with a 1:10,000 dilution of peroxidase-coupled relative antibodies Polyclonal Goat Anti-Rabbit Immunoglobulins HRP, 1:5,000 (Dako, P00448). Antibody–protein complexes were then detected using the Novex ECL Chemiluminescent substrate reagent kit (Thermo Fisher Scientific, WP20005) followed by autoradiography.

### Transwell migration assay

Transwell chambers were used according to manufacturer’s instructions (Falcon, 353097). In brief, both FNCB4 and GN11 cells were grown in complete medium until subconfluence. Cells were then detached and resuspended in SFM. The composition of culture media has been described above. We seeded 20,000 cells/200 μL/condition on the upper side of 8 μm pore membranes and incubated for 12 hours with SFM, SFM+100 μM DAPT, or SFM+1% DMSO. SFM or DMEM supplemented with 2.5% of FBS were placed in the lower chamber accordingly with the experimental design.

After the incubation time, cells in the upper part of the chamber were mechanically removed and cells on the lower side fixed in 70% ethanol at 4°C for 30 minutes before nuclei labeling with Hoechst. Five nonoverlapping regions were imaged per membrane using a ZEISS 20× objective (NA 0.8) mounted on an Axio Imager Z2 light microscope (ZEISS), with nuclei counted using an ImageJ plugin (NIH) and averaged per well.

### Cellular localization of JAG1 protein

Transfected HEK293T cells were permeabilized with 0.1% Triton X-100 (MilliporeSigma, 9036-19-5) and incubated with primary anti-Jagged1 (28H8) and 5% donkey serum (MilliporeSigma, D9663) overnight at 4°C. The following day, we incubated with the secondary antibody (Alexa Fluor 488 goat anti-mouse IgG, Invitrogen, A-11001) 1:1,000 for 1 hour at room temperature, and we stained the ER with the ConA Alexa Fluor 594 conjugated (Invitrogen, C11253) for 1 hour at room temperature at 5 mg/mL.

### Real-time PCR on GN11 and FNCB4 cells

RNA extraction on GN11 and FNCB4 cell lines was performed using the EZNA Total RNA Kit II (Omega Bio-Tek, R6934-02), and the cDNA was reverse-transcribed using the SuperScript III kit (Thermo Fisher Scientific, 18080051). qPCR was carried out on Applied Biosystems 7900HT FasT Real-Time PCR System using exon span–specific TaqMan Gene Expression Assay (Applied Biosystems, 4440040). The following primers were used: *Jag1* (Mm00496202_m1), *Notch1* (Mm00627185_m1), *Notch2* (Mm00203077_m1), *Notch3* (Mm1345646_m1), *Notch4* (Mm00440525_m1), *JAG1* (Hs01070032_m1), *NOTCH1* (Hs01062014_m1), *NOTCH2* (Hs01050702_m1), *NOTCH3* (Hs01128537_m1), *NOTCH4* (Hs00965889_m1), and *18S* (Hs99999901_s1) and *Actb* (Mm00607939) as housekeeping genes. Amperase activation was achieved by heating at 50°C for 2 minutes, before denaturation at 95°C for 20 seconds, followed by 40 cycles of 1 second at 95°C with 20 seconds of extension time at 60°C. Gene expression data were analyzed using SDS 2.4.1 and Data Assist 3.0.1 software (Applied Biosystems). Results were analyzed with the standard ΔC_T_ method and normalized to the expression of the housekeeping genes.

### Zebrafish strains and treatments

Zebrafish (*Danio rerio*) embryos obtained from natural spawning were raised and maintained according to EU regulations on laboratory animals (Directive 2010/63/EU). All experimental protocols were carried out in accordance with relevant guidelines and regulations of Good Animal Practice approved by the institutional and licensing committee IACUC and University of Milan by the Italian Decree of March 4, 2014, n.26. Embryos were staged according to morphological criteria (80). From the epiboly stage (around 6 hpf) embryos were cultured in fish water containing 0.003% 1-phenyl-2-thiourea (MilliporeSigma, 189235) to prevent pigmentation and 0.01% methylene blue to prevent fungal growth.

The zebrafish tg(GnRH3:EGFP), tg(12xnre:mCherry), and tg(*omp^2k^:gap-CFP^rw034^*) lines were used in the present study. The fish strain tg(GnRH3:EGFP) (38, 81) was obtained from Gothilf Lab (Tel Aviv University, Tel Aviv, Israel). The tg(12xnre:mCherry) (40) zebrafish line, reporter for the 12 Notch responsive genes, was provided by the Argenton lab (Padua University, Padua, Italy). Finally, the tg(*omp^2k^:gap-CFP^rw034^*) line (46–48), reporter line for the olfactory axons, was provided by the Merlo lab (University of Turin, Turin, Italy). The *omp^2k^:gap-CFP^rw034^* strains were used to visualize the placodal olfactory neurons and their axons.

Morpholino (MO) antisense oligonucleotide KD strategy (82) was used for downregulation of *jag1a* (GenBank accession number: NM_131861) and *jag1b* (GenBank accession number: NM_131863) expression. MOs were synthesized by Gene Tools. Zebrafish zygotes were injected at the 1- to 2-cell stage with 0.7 and 0.6 pmol/embryo of *jag1a* and *jag1b* MO, respectively. Standard control MO was microinjected at 0.35 or 0.3 pmol/embryo when jag1 MOs were injected alone and 0.75 pmol/embryo for the double jag1 MO injection.

### Probe synthesis and WISH

Antisense probes amplified by PCR from zebrafish total RNA, followed by cloning into pGEM-T Easy Vector (Promega, A1360), and in vitro–transcribed using mMessageMachine (Invitrogen, AM1344). The primers used for PCR amplification are listed in Supplemental Table 1. WISH experiments were performed according to standard protocols (83).

### DAPT treatment

One hundred millimolar stock of the γ-secretase inhibitor DAPT (MilliporeSigma, D5942) was resuspended in DMSO (MilliporeSigma, D8418) and stored at −20°C. Embryos were treated, from 50% epiboly to 48 or 72 hpf with DAPT, diluted to 50 or 100 μM (41), in fish water. Control embryos were treated with 1% DMSO as vehicle.

### Imaging

WISH embryos were mounted in 85% glycerol on a depression slide and acquired with a Leica M205FA equipped with Leica DFC450FC digital camera. Confocal acquisitions of reporter embryos were performed using Nikon Eclipse Ti microscope equipped with a 20× objective. For the in vivo acquisition of GnRH3 fibers and olfactory neurons, the tg(GnRH3:EGFP) and tg*(omp^2k^:gap-CFP^rw034^*) embryos were placed in a 35 mm imaging dish with an Ibidi Polymer Coverslip Bottom (catalog 81156) and covered with 1% low-melting agarose gel (MilliporeSigma, 2070-OP).

### Data availability

The functional studies’ data that support the findings of this study are available on request from the corresponding author. The raw human genetics data are not publicly available due to consent form restrictions.

### Statistics

Statistical analysis was performed with PRISM software 8.0 (GraphPad Software). Normal distribution was determined with the Shapiro-Wilk normality test for all samples before any group analysis. Sample sizes were chosen according to standard practices and are shown in each figure legend.

No randomization method was used to assign samples in the experimental groups or to process data. No study size calculation was performed. No data were excluded from the study.

For each experiment, replicates are described in the figure legends. When normally distributed, data were compared using an unpaired 2-tailed Student’s *t* test or a 1-way ANOVA for multiple comparisons against the control condition, followed by Tukey’s multiple-comparison post hoc test. Data not following normal distribution were analyzed using either a Mann-Whitney *U* test (comparison between 2 experimental groups) followed by a Fisher’s test or Wilcoxon/Kruskal-Wallis test (comparison between 3 or more experimental groups), followed by a Dunn’s post hoc analysis. *P* < 0.05 was considered statistically significant.

### Study approval

#### Zebrafish.

All procedures were performed on zebrafish embryos within 5 days postfertilization according to EU regulation on laboratory animals (Directive 2010/63/EU) and conformed to Italian Legislative Decree no. 2014/26. The zebrafish studies were approved by the Body for the Protection of Animals of the University of Milan (protocol 198283).

#### Human fetuses.

Fetal tissues were made available in accordance with French bylaws (Good Practice Concerning the Conservation, Transformation, and Transportation of Human Tissue to Be Used Therapeutically, published on December 29, 1998). The studies on human fetal tissue were approved by the French agency for biomedical research (Agénce de la Biomédecine, Saint-Denis la Plaine, France, protocol PFS16-002). Nonpathological human fetuses were obtained from pregnancies terminated voluntarily after written informed consent of the parents (Gynaecology Department, Jeanne de Flandre Hospital, Lille, France).

#### Human participants.

The study, in accordance with the Declaration of Helsinki, was approved by the Ethics Committees in Milan (Italy) (GR-2008-1137632) and in Lausanne (Switzerland) (PB_2018-00247; registered on ClinicalTrials.gov as NCT01601171), and all patients or their legal guardians gave written informed consent.

## Author contributions

LC conducted experiments, acquired and analyzed data, and wrote the manuscript; FM and PD performed experiments in zebrafish, analyzed the data, and wrote the manuscript; MA, GEP, and LB conducted genetic experiments and acquired and analyzed data; NS, MLM, AT, WSD, AM, and GG provided reagents; NP, LP, MB, and VV provided clinical and genetic information for the human study and were involved in all aspects of the manuscript preparation; PG and VV designed the study, analyzed data, and wrote the manuscript. All authors read and approved the final manuscript.

## Supplementary Material

Supplemental data

## Figures and Tables

**Table 1:**
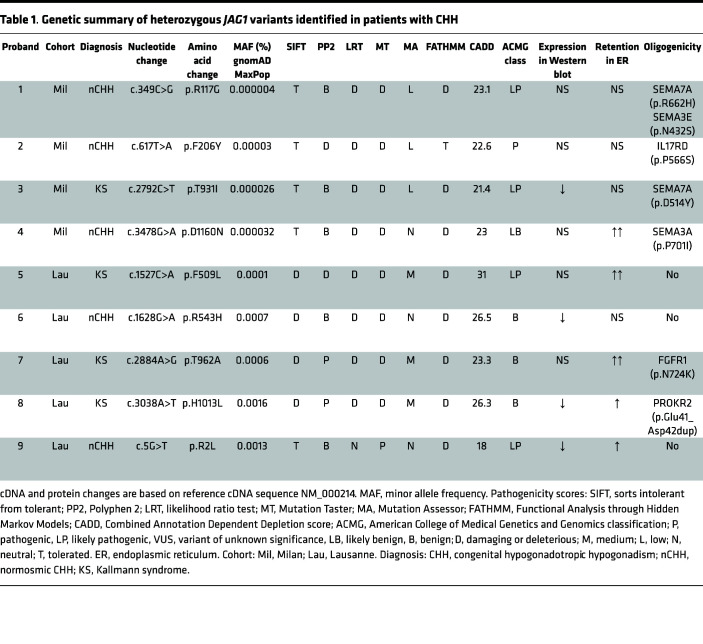
Genetic summary of heterozygous *JAG1* variants identified in patients with CHH

**Table 2:**
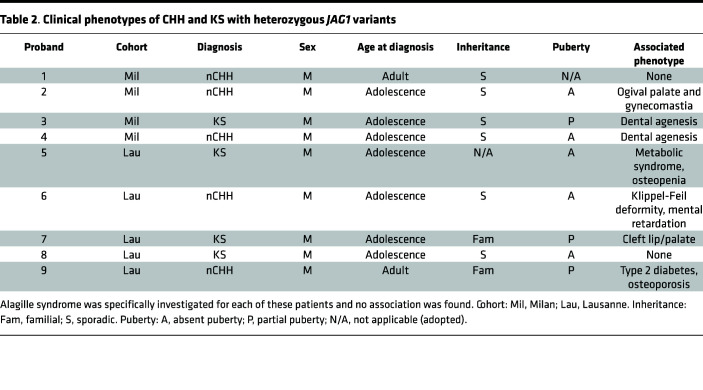
Clinical phenotypes of CHH and KS with heterozygous *JAG1* variants

## Version 1 02/02/2023

In-Press Preview

## Version 2 03/08/2023

Electronic publication
