# Supplementary material for: Defective jagged-1 signaling affects GnRH development and contributes to congenital hypogonadotropic hypogonadism
Source: JCI Insight. 2023 Mar 8;8(5):e161998. doi: 10.1172/jci.insight.161998 (PMC10077483; doi:10.1172/jci.insight.161998)
Supplement: Supplemental data [file jciinsight-8-161998-s082.pdf]

Supplemental material

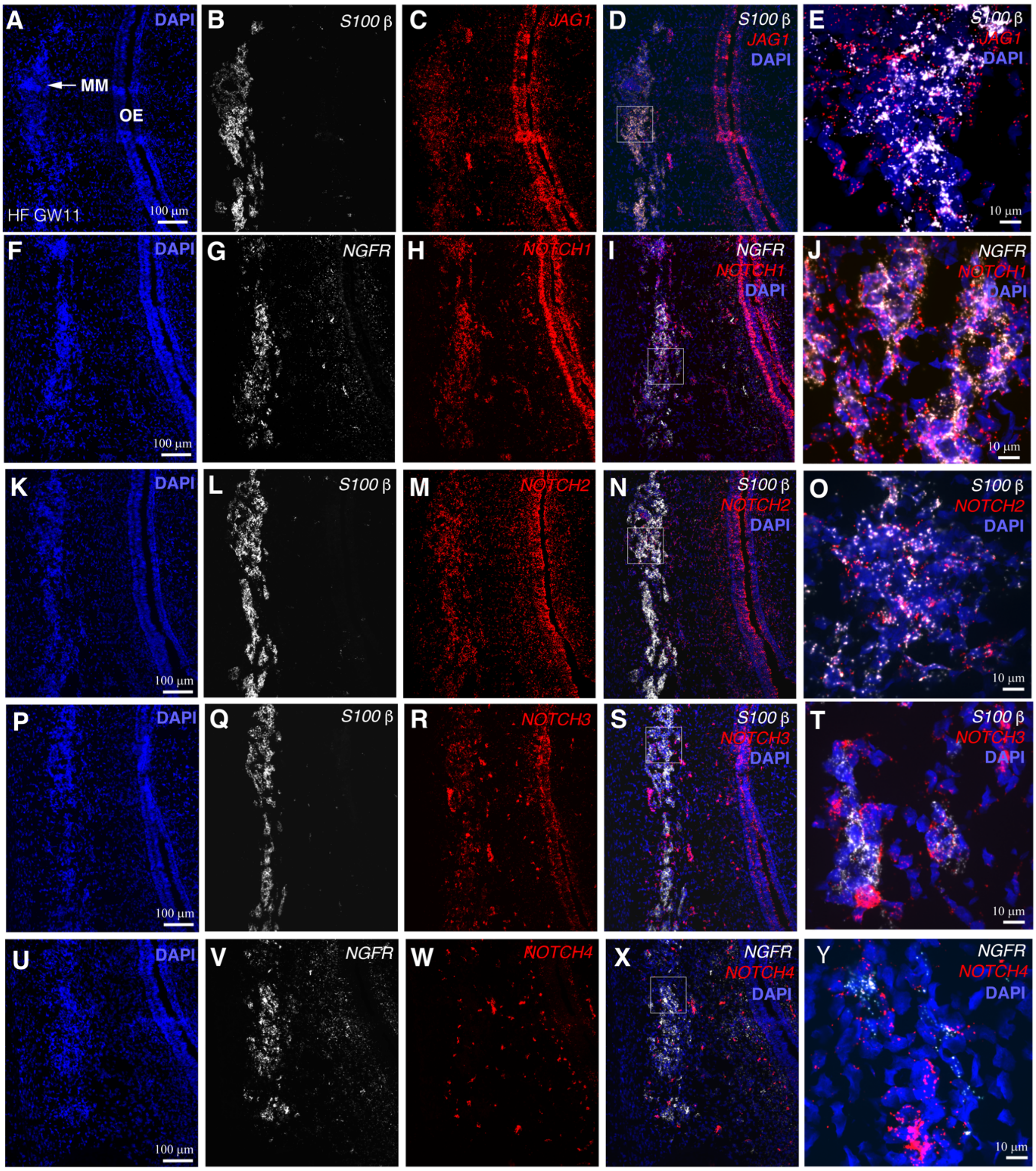

Figure S1: *Jag1* and *Notch1-3* transcripts are expressed in the OECs of the nasal compartment of human foetuses. A-Y, FISH analysis of *JAG1*, *NOTCH1-NOTCH4*, *S100 β* and *NGFR* in a GW 11 human fetus head (coronal view). **E, J, O, T, Y,** Representative single-plane high power confocal micrographs of areas depicted in squared boxes. The experiments have been replicated 3 times with comparable results. OE: olfactory epithelium; MM: migratory mass; HF: human fetus; GW: gestational weeks.

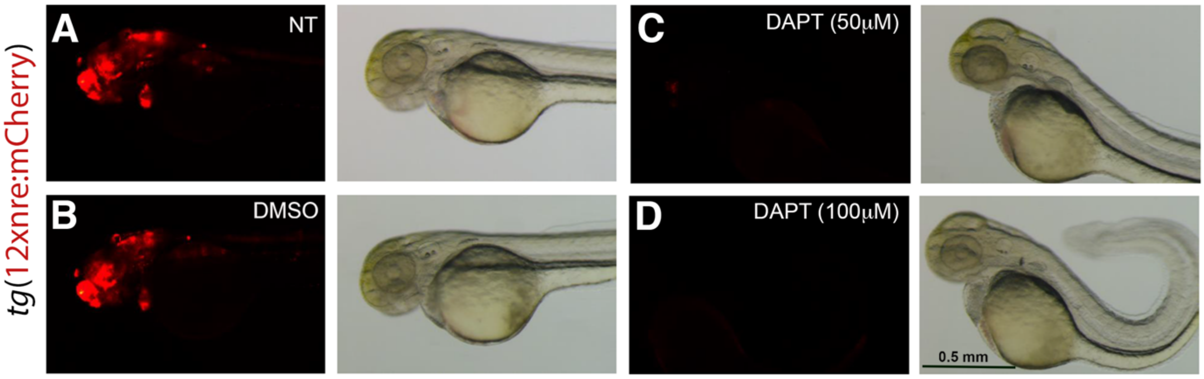

Figure S2: ***DAPT treatment silences the expression of the Notch responsive element*****. A-D** Representative confocal image displaying mCherry expression in the zebrafish reporter line tg(12xnre: mCherry) either untreated (**A**), treated with DMSO (**B**) or with two indicated doses of DAPT (**C, D**) at 48hpf. The treatment was performed 4 times on n = 30 transgenic embryos per condition. Brightfield images show the morphology of the embryos under the different treatment conditions. Scale bar: 0.5 mm.

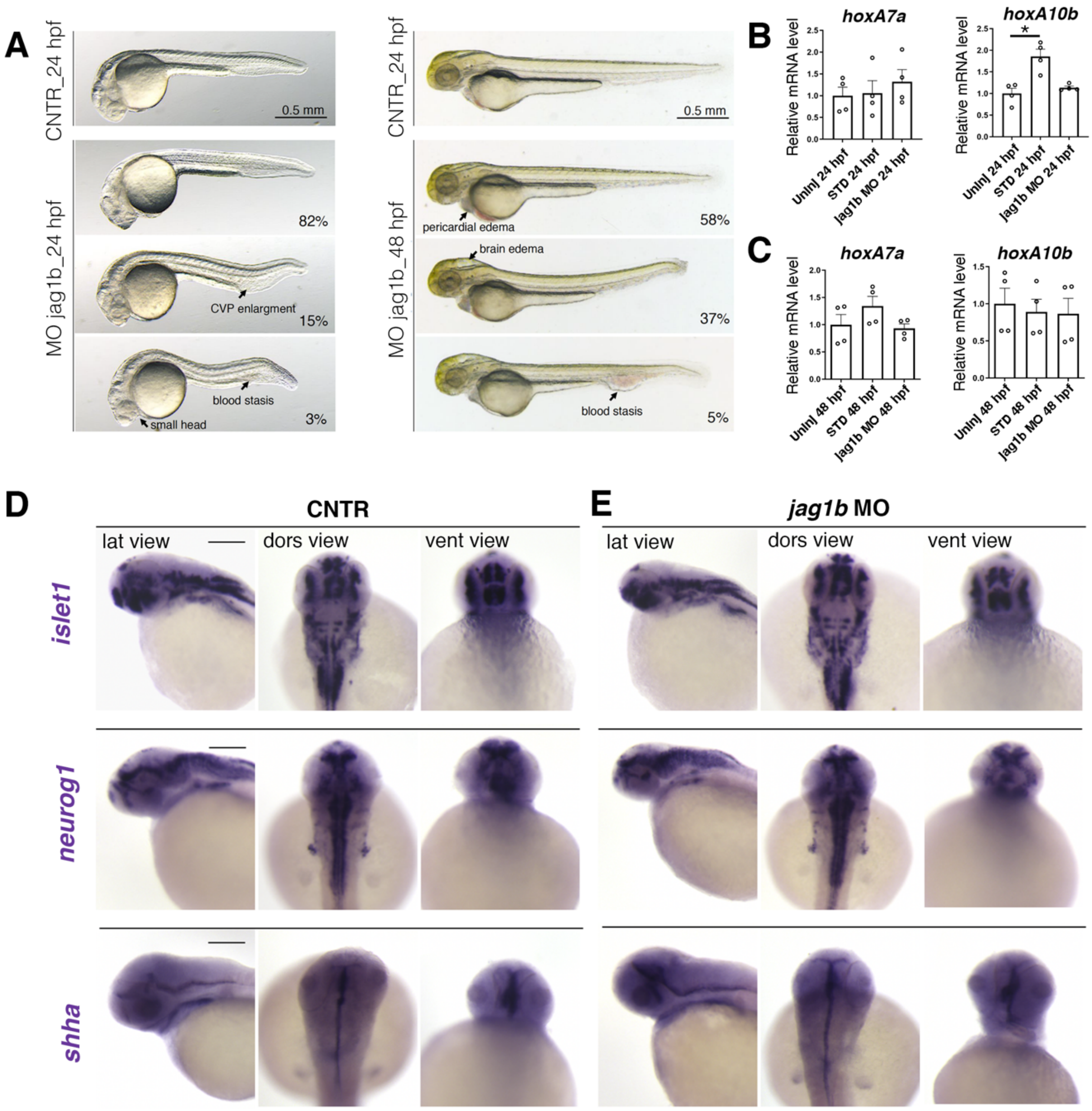

Figure S3. Morphological analysis of *jag1b* morphants. **A,** Representative whole-mount bright-field images of control and MO-injected embryos at 24 hpf and 48 hpf. The experiments were performed 3 times with n = 30 embryos per condition. **B, C,** qRT-PCR analysis showing the relative abundance of *hoxA7a* and *hoxA10b* mRNAs in whole embryos injected with jag1b MO at 24hpf and 48hpf, respectively. Data represent mean ± SEM. **D**, **E**, Whole-mount in situ hybridization for ISL LIM homeobox 1 (*islet1)*, neurogenin 1 (*neurog1*) and sonic hedgehog signaling molecule a (*shha*) in the forebrain of control (**D**) and *jag1b* morphants (**E**) at 48 hpf. Representative images refer to lateral, dorsal and ventral views. Scale bars in **D** and **E**: 200 μm

GnRH3 Fw 5'AGCATGGAGTGGAAAGGAAG 3'	GnRH3 Rv 5'AGCCCATCTGTTCCTTCAGT 3'
Jag1a Fw 5'ATGGGAAGGAGCAACGTGTA 3'	Jag1a Rw 5'AAGGTGAAGGTGACGTTTGC 3'
Jag1b Fw 5'GAGTGTCAGTCTTCTCCCTG 3'	Jag1b Rw 5'TCAGTTTTGGGGCTTGGTAT 3'
Notch1a Fw 5'AGTAACGGCGGCGTGTGTCA 3'	Notch1a Rw 5'TGATGCCACTGAAGCCCGCA 3'
Notch1b Fw 5'ATCACGCCGGCTCCTAAGGT 3'	Notch1b Rw 5'TGCAGAGCCGCCGAACAGTT 3'
Notch2 Fw 5'ATAACGGCCGCTGTGACCCA 3'	Notch2 Rw 5'TCCGTGTTGCAGCCCTGGTT 3'
Notch3 Fw 5'AGCCCATCCTGCAGCCAACA-3'	Notch3 Rv 5'TGGCTGTGAAGTTGCTGCGG 3'
Islet1 Fw 5' AACCTGAGAAAACAACCCGC	Islet1 Rw 5' TCAGAAGCGGCCCTAAATGA
Neurog1 Fw 5' CTGACGACACAAAGCTGACC	Neurog1 Rw 5' TCTAACGGGGTTCTGGTCAC
Shha Fw 5' ACATCGGGTGAGCTAGAGTG	Shha Rw 5' GGATGTGAACCGAAGCGTTT

Table S1: list of primers used for riboprobes' synthesis.
